# Keratoconus: The Local Manifestation of a Systemic Disease?

**DOI:** 10.3390/jcm14134587

**Published:** 2025-06-28

**Authors:** Matteo Pederzolli, Federico Procopio, Beatrice Tombolini, Simone Marra, Massimo De Micheli, Francesco Bandello, Giulio Ferrari

**Affiliations:** 1 Eye Repair Lab, Division of Neuroscience, IRCCS San Raffaele Scientific Institute, 20132 Milan, Italy; 2 Cornea and Ocular Surface Disease Service, Ophthalmology Unit, IRCCS San Raffaele Scientific Institute, 20132 Milan, Italy; 3 Department of Ophthalmology, Vita-Salute San Raffaele University, 20132 Milan, Italy; 4 Ophthalmology Department, Ospedale di Circolo e Fondazione Macchi, ASST Sette Laghi, 21100 Varese, Italy; 5 Ophthalmology Unit, Ospedale Maggiore di Lodi, 21100 Lodi, Italy

**Keywords:** keratoconus, systemic, KC, vitamin D, Vit D, review

## Abstract

Keratoconus (KC) is the most common ectatic corneal disease. In this review, the systemic aspects of the disease are discussed, including patient age, genetics, systemic inflammatory status and immune system dysregulation, atopy and eye rubbing, systemic metabolism, the metabolism of micronutrients (including vitamin D), and hormonal balance. The association between KC and metabolic diseases, collagen diseases, and psychiatric conditions is also considered. The evidence that is currently available strongly suggests a systemic predisposition toward KC. The understanding that KC may be the local manifestation of a systemic disease could allow earlier detection/prevention and pave the way for research into new treatments addressing the pathogenetic foundations of KC, rather than limiting intervention to the corneal disease.

## 1. Introduction

Keratoconus (KC) is a bilateral ectatic corneal disorder that results in progressive thinning and steepening of the cornea, irregular astigmatism, and decreased visual acuity [1,2,3].

The 2015 Global Consensus on KC and Ectatic Diseases reached an agreement on considering KC a disease of many components: a genetic disorder, a biochemical disorder, a biomechanical disorder, and an environmental disorder [2].

In the past decades, evidence has accumulated that links this corneal disease to a series of systemic associated/predisposing factors. This is leading us to reconsider our paradigm of KC as an exclusively corneal disease. Interestingly, many of the diseases associated with KC show increased levels of systemic inflammation. In this vein, while KC used to be defined as a “non-inflammatory” corneal degeneration [3,4,5,6], such a concept has been revisited, as the role of inflammation, both local and systemic, has been acknowledged in the pathogenesis of KC [7,8,9,10].

In this review, we aim to review existing evidence supporting the hypothesis that KC is the ocular manifestation of a systemic disease.

## 2. Genetics

A first clue about KC not being—at least exclusively—caused by strictly local factors is the identification of various genetic alterations involving oxidative stress, extracellular matrix composition, and inflammation.

First of all, the existence of a genetic component in KC is supported by observations on family history: up to 20% of patients report a positive family history [11], and first-degree relatives of patients with KC have a much higher (up to 67%) risk of having KC [12]. In addition, consanguinity has been described as a risk factor for KC [13], and monozygotic twins have a similar predisposition toward the disease [14]. Sporadic, mitochondrial, autosomal dominant, and autosomal recessive traits have been described in association with KC; non-coding genes have also been hypothesized to play a role in KC pathogenesis [15,16,17,18]. Specific genes that have been associated with KC through candidate gene analysis include Visual System Homebox 1 (VSX1), Superoxide Dismutase 1 (SOD1), Zinc Finger Protein 469 (ZNF469), and collagen genes such as COL4A1/2/3/4 and COL8A1/2 [19,20,21,22,23,24,25,26,27,28,29,30,31]. Genome-wide analysis studies have found relevant associations of KC with many genetic variants, although with varying degrees of evidence, such as IL1B, TGF1B, MIR184, DOCK9, LOX, CAST, and single nucleotide polymorphisms of HGF and RAB3GAP1 [32,33,34,35,36,37,38,39,40,41,42,43,44,45,46,47,48,49,50,51,52].

Trisomy 21 (Down syndrome) is a striking example of how systemic predisposition on a genetic basis may interact with environmental factors in promoting KC. The reported prevalence of KC in patients with Down syndrome is variable due to differences in patient age, ethnicity, sample size, diagnostic criteria, and the type of technology used for diagnosis [53,54]. In general, the corneas of patients with Down syndrome appear thinner and steeper and present more aberrations [54,55]. Recently, KC was diagnosed in 26.3–26.9% of patients with Down syndrome based on corneal tomography [55]. When the proportions with KC and suspect KC are combined, the prevalence reaches 71.3% [54]. A nationwide claims database in the USA noted that individuals with Down syndrome had a sixfold increase in odds of having KC [56].

A strong eye-rubbing habit has been related to an increased risk of developing KC in patients with Down syndrome [55,57]. However, other studies have found no association between eye-rubbing and the severity of ectatic changes in the cornea in patients with Down syndrome [58,59].

Chromosome 21 hosts both collagen VI genes (COL6A1 and A2) and SOD1; these genes have been implicated in collagen matrix alterations and increased oxidative stress in patients with Down syndrome [60,61,62,63,64].

Consequently, it is suspected that eye rubbing, collagen structure, and oxidative stress all contribute to the higher risk of KC in patients with Down syndrome, even though their relative weight cannot be determined at the moment.

## 3. Systemic Inflammatory Pathways and Innate Immunity

Systemic inflammation seems to play a pivotal role in the pathogenesis of KC.

From an epidemiological point of view, several systemic inflammatory diseases have been found to be associated with KC, such as rheumatoid arthritis, ulcerative colitis, autoimmune chronic active hepatitis, and irritable bowel syndrome [65,66], although evidence is not unequivocal [67].

Numerous studies have found a correlation between systemic markers of inflammation and KC.

Altered redox metabolism has been demonstrated in the cornea of patients with KC [68,69,70,71], which induces mitochondrial DNA damage [72]. It is also known that, in the cornea of patients with KC, there are reduced levels of nonenzymatic (glutathione) [71,73,74] and enzymatic antioxidants [73,75,76,77].

The increased oxidative stress in the cornea is hypothesized to lead to oxidative damage, with increased extracellular matrix degradation eventually inducing corneal thinning [78,79]. Interestingly, the oxidative metabolism of patients with KC has been found to be altered not only at the ocular surface level but also systemically, in terms of higher levels of ROS, reduced glutathione concentration, and reduced activity of antioxidant-acting enzymes [80,81,82].

The arachidonic acid pathway has also been studied in association with KC: elevated serum levels of prostaglandins (F2-alpha, A2, and E2) as well as 5-hydroxyeicosatetraenoic acid are found in patients with KC [83,84].

Other inflammatory mediators, such as IL-6, TNF-α, and MMP-9, are increased both in the tears and the serum of patients with KC [85,86,87,88,89]; elevated concentrations of interleukin-8 (IL-8) were found both in corneal cells and blood samples from patients with KC [90]; and an increased neutrophil-to-lymphocyte ratio (a marker of systemic inflammation) was found to be associated with progressive KC, once again prompting the suggestion that KC may be part of a systemic inflammatory disorder [91].

To explain such a pro-inflammatory status in patients with KC, a compelling line of research is currently focusing on innate immunity hyperactivation: toll-like receptors 2 and 4 (TLR2 and TLR4) show increased expression both in conjunctival cells and monocytes/neutrophils of patients with KC compared to healthy individuals [92,93]. Such overexpression also correlates with tear and serum levels of inflammatory mediators and parameters of KC severity and is inversely proportional to tear and serum lactoferrin levels [94].

## 4. Atopy and Eye Rubbing 

On the other hand, the association between KC and atopy, a condition primarily characterized by a hyperactivation of acquired (rather than innate) immunity, has been studied extensively and is consisently corroborated by additional evidence [95,96,97]. A recent meta-analysis, for example, showed that patients with allergic rhinitis are more likely to develop KC [98]. Moreover, a causal role of atopic dermatitis in the development of KC has been reported [99]. Most importantly, allergic conjunctivitis is often found in patients with KC [100,101,102,103]; this points to the usefulness of screening for KC during the management of patients with allergic conjunctivitis. A comparison of the proteomic profile in the tears of patients with KC and allergic conjunctivitis pointed to an association between the two conditions at a molecular level [104].

The comorbidity of KC and atopy is further demonstrated by the association between vernal keratoconjunctivitis and KC [102,105]. A significant correlation has been shown between the severity of KC and vernal keratoconjunctivitis [102]. On the other hand, a study conducted in Germany recently failed to show an association between KC and atopy [106].

It is important to note that a state of chronic allergic reaction of the ocular surface—and thus ocular itching—may lead to eye rubbing. The association between eye rubbing and KC emerges clearly in almost all studies carried out on a wide variety of populations [107,108,109]. When tightly controlled, a significant proportion of patients show no disease progression at a three-year follow-up following cessation of eye rubbing, without any need for further intervention [110]. It should also be noted that eye rubbing sometimes represents an addictive behavior in patients with KC [111]. A Chinese study described gene–environment interactions between single nucleotide polymorphisms (rs26515, rs27991, and rs9314177) in the CAST gene (which has been associated with KC) and eye rubbing [35,112].

The exact mechanism by which eye rubbing leads to the development or progression of KC has not been entirely elucidated. It was thought that the cornea of these patients is structurally weaker than normal and rubbing easily promotes its deformation [113]. A recent study showed a strong association between KC, eye rubbing, and an elevated concentration of Langerhans cells in the central cornea [114]. Despite the evidence, KC patients seem to show little awareness of the importance of avoiding this conduct [115]. It is our opinion that the first approach to KC must be thorough patient information and the reduction of trigger stimuli at a local and potentially systemic level (notably, by treating atopic conditions). 

## 5. Vitamin D: A Modulator of Systemic Inflammation

Vitamin D (Vit D) is a pleiotropic fat-soluble prohormone. Perhaps its most commonly known function is immune system modulation: Vit D can act on both innate and adaptive immune responses [116].

As discussed so far, both types of immune response have been implicated in KC pathogenesis, which makes Vit D a factor of high interest.

Furthermore, it has been shown that Vit D plays a central role in corneal homeostasis [117,118,119,120,121,122]. Briefly, Vit D supports epithelial barrier function and endothelial cell survival [117,118,119,120], and it modulates inflammation [121,122] by reducing inflammatory mediators (cytokines, prostaglandins) [123]. Vitamin D can enter the cornea either via tear fluid and aqueous humor [67,124], or it can be synthesized de novo in limbal epithelium after UVB radiation exposure [119,125].

Accumulating evidence supports the hypothesis that Vit D may participate in KC pathogenesis [3,11,126,127,128,129,130,131,132,133,134,135,136,137,138,139,140,141,142]. KC may coexist with conditions associated with Vit D deficiency, including systemic affections (i.e., atopy, asthma, obstructive sleep apnea, Down syndrome, and heart valve and thyroid diseases) [130,131,132,133,134], adolescence, and pregnancy [3,11,135,136,137,138,139]. A 1938 preclinical study on animal models (which had many limitations in light of current knowledge) showed that a Vit D-deficient diet may lead to KC-like ocular alterations [143]. A subsequent 1939 pilot study found that vitamin D supplementation could arrest and even reverse KC [126]. More recently, patients affected by KC were found to have lower serum Vit D levels (<10 ng/mL) [127,128,129]; interestingly, the same has occasionally been reported for patients with myopia [117,144]. Furthermore, Vit D levels have been found to be lower in progressive rather than nonprogressive KC, although there is no unanimous consensus on this point [141].

Our group evaluated the clinical and biological impact of the administration of Vit D in patients with KC and Vit D insufficiency (<30 ng/mL). The first version of the study [145] included twenty adolescent patients who were supplemented for 6 months and followed up for 12 months. Interestingly, 60% of patients (72% of eyes) showed topographic stabilization of KC development (Kmax progression < 1D) after 12 months. Overall, TCT, Kmax, and BSCVA rates remained constant over the observation period. This study confirmed that patients with KC show downregulated vitamin D transport and activation [141]; since Vit D upregulates its own metabolism, supplementation increased both Vit D transport (by VDBP) and Vit D activation (by CYP27A1 and MMP-9/TIMP-1) [145]. Systemic collagen degradation was also found to decrease in the study population.

In 2025, we updated our findings with data obtained by including 20 additional patients [146]. The mitigating effect of Vit D on KC progression and systemic collagen turnover was confirmed in this 40-patient study. Additionally, RNA sequencing on peripheral blood monocytic cells obtained at baseline and after Vit D supplementation revealed a modulation in systemic inflammation and changes in platelet count and/or activity. Notably, RNA transcription patterns were found to be relatively coherent among patients showing KC stabilization and incoherent among non-responders, which points to different systemic predispositions towards Vit D response.

Overall, such data suggest that vitamin D supplementation could be a useful intervention in KC. These preliminary findings will require further confirmation by randomized clinical trials.

## 6. Other Micronutrients with a Possible Role in KC

Vitamin A (Vit A), whose deficiency is associated with pathologies such as keratomalacia and xerophthalmia, has been found to play a role in the development of KC. A 1939 study supplemented Vit A to Vit A-deprived rats; after a few days, the development of KC was observed [147]. It has also been observed that retinoic acid supplementation promotes corneal cross-linking (CXL) by increasing the expression of transglutaminase 2 (TG-2) in the corneal epithelium [148].

In an integrated analysis conducted on mouse models, it was observed that a significantly higher percentage of mice with KC had Vit A deficiency (VAD) than controls, and among KC mice, VAD was significantly higher in females [149]. A transcriptomic analysis found that genes that were more upregulated in female mice with VAD compared to female controls were mainly related to the immune response and were involved in the JAK-STAT pathway. In contrast, the differentially expressed genes between VAD and control male mice pertained to the estrogen signaling pathway [149].

Patients with KC have lower blood levels of riboflavin (B2) than healthy controls; this renders riboflavin a candidate risk factor for KC development. Blood levels of homocysteine, folic acid, and vitamin B12 have also been studied but showed no significant differences between patients with KC and the control group [150].

Serum levels of copper (CU), selenium (Se), and zinc (Zn) are lower in patients with KC compared to the general population [130,151,152]. In particular, copper (Cu) participates in collagen CXL and is known for its antioxidant activity [80,81,152,153,154]. Patients affected by KC display an altered copper metabolism [130,142,145,151,155], presenting reduced systemic levels of copper relative to healthy individuals [130,151]. Moreover, decreased copper levels have been detected in the central cornea of these patients [81,154,155,156]. Our group found enhanced copper availability after Vit D supplementation and hypothesized that copper metabolism alterations may contribute to KC progression despite normal Vit D levels [145].

Iron is also suspected to play a role in the pathogenesis of KC. High levels of iron are found (together with copper) in Fleischer’s corneal ring [155,156], even though no significant differences in serum iron levels have been observed in patients with KC [151]. Patients with KC exhibit reduced levels of iron-binding proteins, such as serotransferrin and lactoferrin, in both their tears and corneal epithelium, indicating local disruption in iron homeostasis within the KC cornea [141,157,158]. Polymorphisms of the transferrin gene have been identified as risk factors for KC. Altered iron metabolism may increase KC risk not only because it reduces the function of CXL enzymes but also because it generates reactive oxygen species through the Fenton reaction, leading to oxidative stress, which is known to be a risk factor for the development of KC [159,160]. Ferroptosis has also been implicated in KC, with recent studies identifying six ferroptosis-related genes differentially expressed in patients with KC using mathematical algorithms [161].

## 7. The Ocular Surface Microbiota

The gut–eye axis is an emerging concept that suggests that the gut microbiome can influence systemic inflammation and potentially affect the eyes [162]. While there is a lack of specific studies investigating intestinal microbiota alterations in patients with KC, a growing body of research is exploring the role of the ocular surface microbiota in this condition [162,163,164].

This initial body of evidence is mainly focused on the relative prevalence of different classes of bacteria in keratoconic and healthy corneas, but it remains ambiguous [165].

## 8. Does Systemic Metabolism Impact KC?

The Krebs cycle, or the tricarboxylic acid cycle, is believed to be involved in the development of KC, as increased lactate production has been found in KC both locally and systemically [166,167,168]. Abundant lactate production implies anaerobic respiration, which can then result in a reduction in intracellular pH, oxidative stress, and apoptosis [169].

In vitro models of KC found glucose metabolism to be upregulated, a finding that was later confirmed by an analysis carried out on the tears of patients with KC; anaerobic glycolysis appears to be the most affected pathway, as lactate concentrations higher than those of pyruvate are found [170,171]. In addition, it is well known that sex hormones can alter glucose metabolism (see later), and KC develops right at pubertal age [172,173].

Following in vitro CXL, there is a shift to aerobic respiration, with a significant increase in ATP production [166]. The lactate-to-malate ratio has also been proposed as an indicator of oxidative stress [71,168]. However, it is surprising to note that in the tears of patients with KC, there is increased production of ATP, malate, and malonyl-CoA [74].

Urea cycle metabolism has also been studied in relation to KC. Stimulation of the cells of patients with KC with dehydroepiandrosterone (DHEA), which is increased in the circulation of patients with KC, increases the urea cycle by altering the bioavailability of precursors necessary for collagen synthesis such as proline and hydroxyproline [84,173,174]. This finding is supported by the fact that reduced levels of ornithine and increased levels of aspartate have been found in the tear film of patients with KC [74].

Regarding acid metabolism, one study showed a significant reduction in both saturated and unsaturated fats in KC corneas [175]. This may be explained by the fact that patients with KC have reduced levels of malonyl CoA, a key precursor for fatty acid synthesis [74,176].

Docosahexaenoic acid (DHA) administration was found to significantly improve antioxidant capacity and reduce the expression of inflammatory cytokines. Such evidence, obtained through a preliminary study, corroborates the possible utility of DHA administration to target KC [177]. 

## 9. Association with Metabolic Disorders

Higher body mass index values are consistently associated with KC, suggesting that obesity is a risk factor for KC development [178,179,180]. Increased body mass index also correlates with an increased frequency of eye rubbing, which increases the risk of KC manifestation or progression [181]. KC is frequently noted in individuals with obstructive sleep apnea (OSA, a condition associated with obesity), possibly due to floppy eyelid syndrome, which is also strongly associated with KC [182,183]. A meta-analysis found that KC was associated with a 1.87 times higher risk of OSA compared to controls [184]. Furthermore, there is a significant increase in corneal steepening among patients diagnosed with severe OSA [185].

Diabetes could potentially serve as a protective factor in KC [186], as it has been proposed that it facilitates collagen CXL in the cornea [187]. Nonetheless, further studies indicated no significant correlation between KC risk and diabetes [188,189,190]. However, it was noticed that individuals with diabetes exhibited reduced odds of advancing their KC to a more severe stage [191].

## 10. Hormonal Balance and KC Risk

The role of thyroid hormones in the development of KC is still being investigated. A recent analysis demonstrated a higher prevalence of KC in patients with thyroid dysfunction [192], particularly among females [193]. Additionally, such patients showed increased maximum simulated keratometry value and thinner corneas [193]. This association has been confirmed by different authors, who have found a higher incidence of KC in the female sex and in association with hyperthyroidism [192,194]. On the contrary, no association was found between congenital hypothyroidism diagnosed and treated from newborn age and KC [195], and the severity of KC does not seem to change depending on the presence of hypothyroidism [196].

From a molecular perspective, elevated thyroxine values were described in the tears of patients with KC [197,198] and in the aqueous humor [199]. T4 receptor expression was also elevated in keratinocytes of patients with KC compared to controls, suggesting an active role of T4 in the development of KC [198]. In vitro, thyroxine was found to increase collagen I expression in fibroblasts of patients with KC but not in keratinocytes, while it had no effect on the production of transforming growth factor β1 (TGF-β1) or collagen V in either cell population, thus making it difficult to think that increased thyroxine concentration alone could play a role in the development of KC [200].

It should be noted that Hashimoto thyroiditis is an autoimmune disease. Therefore, regardless of the changes in thyroid hormone imbalance, it is possible that increased KC prevalence is a consequence of the dysregulated systemic immune response. In this sense, a statistically significant association of KC with Hashimoto’s thyroiditis, specifically, has also been described [201].

Data on the influence of sex hormones in KC are heterogeneous, and it is not yet certain how much impact they have on KC and which molecular pathways are involved [202]. Gender prevalence in KC is uncertain and may vary depending on geographical location [56,203,204,205]. A potential role of sex hormones in KC development has been postulated from the observation that KC typically develops during puberty, with stabilization around 40 years of age, when relevant hormonal changes occur [11,206]. Estrogen and androgen receptor expression is higher in keratinocytes of patients with KC compared with controls, while the level of progesterone receptor expression is lower [207,208]. Moreover, salivary and blood levels of estriol and estrone were found to be reduced in patients with KC, while dehydroepiandrosterone sulfate levels were increased [166,167,209]. Another study found reduced testosterone levels in the plasma of both male and female patients with KC [210]. A role for gonadotropins is also possible, as an altered luteinizing hormone/follicle-stimulating hormone ratio has been observed in patients with KC [211].

In women with KC, serum prolactin levels are found to be elevated [212]. A role of prolactin-induced protein as a marker of KC progression has also been proposed, as its levels are found to be altered in the tears, aqueous humor, plasma, and saliva of patients with KC [199,209].

Cases of development or progression of KC during or immediately after pregnancy have also been described [136,137]. Finally, it is interesting to highlight the case of KC progression following the administration of gender-affirming hormone therapy in a male-to-female transgender patient [213].

In conclusion, although the hypotheses revolving around a hormonal involvement in the pathogenesis of KC are intriguing, currently available data show an inconsistent level of evidence.

## 11. Association with Collagen Diseases

KC has been related to several congenital connective tissue diseases, notably mitral valve prolapse (MVP) and Ehlers–Danlos syndrome (EDS). Early studies observed the association between MVP and KC [214]. Further research revealed that 38–58% of individuals suffering from KC also exhibited MPV [214,215]. However, in later studies, much lower—but still notable—proportions of 11–13.4% of patients with MVP were found to have KC [216,217]. Interestingly, a study involving individuals with severe KC who also had corneal hydrops found that two-thirds of the patients had MVP [218]. A retrospective nationwide matched cohort study in Taiwan observed that patients over 40 years old with KC exhibited an almost twofold MPV risk compared to age-matched individuals [219].

Ehlers–Danlos syndrome (EDS) is characterized by skin hyperextensibility, joint hypermobility, and tissue fragility [220]. Metacarpophalangeal and wrist joint hypermobility is five times higher in patients with KC, emphasizing the potential risk of the disease in those with EDS [221]. Genetic variations were identified among several genes shared between the two disorders, such as COL5A1, TNXB, ZNF469, and COL12A1 [222].

Marfan syndrome is frequently mentioned as related to KC, but a large review of affected individuals did not confirm this finding [223].

## 12. Possible Association with Psychiatric Conditions

KC has been occasionally linked to Tourette’s disease, a psychiatric disorder that involves severe obsessive-compulsive behaviors and involuntary eye rubbing [224,225]. An association between ADHD and KC has also been described, although the severity of ADHD did not correlate with the severity of KC [226]. The correlation between KC and depression is debated [56,66,227,228,229].

In general, data on the relationship between KC and psychiatric conditions are limited, and, although compelling, these associations largely need to be proven by stronger evidence.

## 13. Conclusions

**Figure 1:**
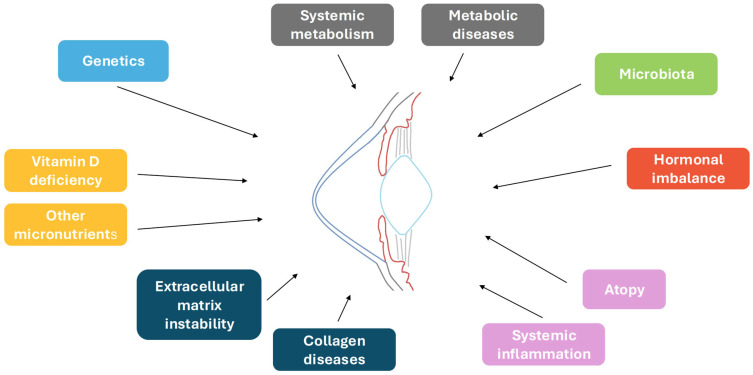
Summary of possible systemic alterations predisposing to keratoconus development. These factors may act independently or, as in the case of systemic inflammation, they may influence one another and work synergistically with local factors (such as eye rubbing), leading to the ectatic phenotype.

In conclusion, although many of the presented lines of study need to be consolidated, growing evidence seems to converge toward a systemic basis for KC (Figure 1). Based on the reviewed data, we suggest that the time is coming for a paradigm shift in the definition of KC. We propose that KC should be considered the corneal manifestation of a generalized disease or the final common corneal phenotype where constitutional (and, possibly, genetic) predisposition plays a relevant role, possibly exacerbated by local factors. In this vein, we could look at KC in the same way as we look at pseudo-exfoliation syndrome, which is mainly known to cause ocular complications but is associated with a generalized impairment of the extracellular matrix [230]. 

There are practical implications of this new definition of KC. First, patients with KC should be routinely investigated to search for systemic comorbidities, and second, efforts should be made to test treatments aimed at controlling inflammation and extracellular matrix impairment.

Further high-quality data are needed to confirm our interpretation.
